# Optimization of an erythroid culture system to reduce the cost of *in vitro* production of red blood cells

**DOI:** 10.1016/j.mex.2018.11.018

**Published:** 2018-11-30

**Authors:** Saiphon Poldee, Chanatip Metheetrairut, Sutthinee Nugoolsuksiri, Jan Frayne, Kongtana Trakarnsanga

**Affiliations:** a Department of Biochemistry, Faculty of Medicine Siriraj Hospital, Mahidol University, Bangkok, 10700, Thailand; b School of Biochemistry, Faculty of Biomedical Sciences, University of Bristol, Bristol, BS8 1TD, UK

**Keywords:** A 3-stage erythroid culture system, *In vitro* erythropoiesis, Insulin, Heparin, Erythroid culture system

## Abstract

*In vitro* generation of red blood cells has become a goal for scientists globally. Directly, *in vitro*-generated red blood cells (RBCs) may close the gap between blood supply obtained through blood donation and high demand for therapeutic uses. In addition, the cells obtained can be used as a model for haematologic disorders to allow the study of their pathophysiology and novel treatment discovery. For those reasons, a number of RBC culture systems have been established and shown to be successful; however, the cost of each millilitre of packed RBC is still extremely high. In order to reduce the cost, we aim to see if we can reduce the number of factors used in the existing culture system. In this study, we examined how well haematopoietic stem cells proliferate and differentiate into mature red blood cells with modified culture system.

• Absence of extra heparin or insulin or both from the erythroid differentiation media did not affect haematopoietic stem cell proliferation and differentiation.

Therefore, we show that the cost and complexity of erythroid culture can be reduced, which may improve the feasibility of *in vitro* generation of red blood cells.

## Graphical abstract

**Figure:**
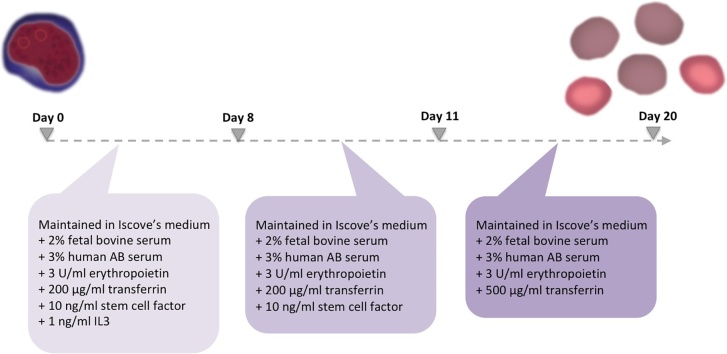

**Specifications Table**

**Table:** 

Subject Area	• *Biochemistry, Genetics and Molecular Biology*
More specific subject area:	*Erythropoiesis*
Method name:	A 3-stage erythroid culture system
Name and reference of original method	A 3-stage erythroid culture system from: Griffiths RE, Kupzig S, Cogan N, Mankelow TJ, Betin VM, Trakarnsanga K, et al. Maturing reticulocytes internalize plasma membrane in glycophorin A-containing vesicles that fuse with autophagosomes before exocytosis. Blood. 2012;119(26):6296-306.
Resource availability	NA

## Method details

Blood shortage is an important health care problem around the world, especially in developing countries. At present, blood donation is the only way that provides blood supplies for transfusion. It has been reported by WHO that 110 million blood units are collected globally every year, however shortage of blood supply still occurs in several countries especially in developing countries [1]. In some situations, such as during war or an epidemic, the demand for blood outweighs supply and patients perish.

However, even in situations when blood supply is adequate, problems still arise. The proportion of different blood groups varies between people in different countries and of different ethnicities. Those individuals with rare blood groups have a higher risk of suitable blood not being available. Also, patients with diseases such as thalassemia, renal failure and cancer usually suffer chronic anemia. Most will need serial blood transfusions, which can stimulate immunity to minor blood group antigens, and cause ‘transfusion reaction’. This reaction can increase morbidity and mortality of patients. Therefore, it is important to have fully compatible blood for transfusion. Moreover, there are many infectious agents that can be transmitted *via* blood, such as hepatitis B virus, HIV and prions. Thus, extensive efforts are made to check that every unit of blood is safe from such agents – a procedure that is not 100% safe, and is also expensive. Hence, to overcome many of these problems, efforts are being made globally to develop system to generate red blood cells (RBCs) *in vitro* from stem cell sources. Different culture systems are used and varying amounts of mature RBCs are produced [[2], [3], [4], [5], [6], [7]]. However, systems to generate RBCs *in vitro* have not yet achieved a clinical grade transfusion product due to the complicated processes involved. RBC production requires many cytokines and growth factors during the various developmental stages, which results in high cost of production. In 2012, Griffiths et al. has developed a culture system that can promote >10^5^ fold expansion of erythroid cells derived from haematopoietic stem cells in adult peripheral blood which achieved very high enucleation rate (up to 95%) [8]. The culture media in this culture system contains several cytokines and growth factors including heparin and insulin. Heparin is a factor that promotes stem cell proliferation and haematopoiesis [9,10]. Insulin is a growth factor that helps regulate cellular metabolism and promote growth and differentiation of several cell types including erythroid cells [[11], [12], [13], [14], [15]]. The aim of this study was to optimize this existing erythroid culture system in order to reduce the cost of production. We show that absence of heparin and insulin from the existing erythroid differentiation media did not affect hematopoietic stem cell proliferation and differentiation; therefore the cost and complexity of erythroid culture can be reduced, which may improve the feasibility of *in vitro* generation of red blood cells.

## Material and methods

### Isolation of adult CD34+ cells

Leucocyte reduction system (LRS) cones were obtained from healthy donors with written informed consent for research use in accordance with the Declaration of Helsinki and approved by Siriraj Institutional Review Board (COA no. 438/2559). Three LRS cones from 3 donors were used for each part of the experiment (n = 3). CD34+ cells were isolated from the peripheral blood mononuclear cell fraction using a MiniMacs direct CD34+ progenitor cell isolation kit (Miltenyi Biotech Ltd.) following the manufacturer’s instructions, as briefly stated below.

1 Blood samples were first diluted with Hanks’ Balanced salt solution (HBSS)(Sigma-Aldrich) containing 0.6% (v/v) citrate dextrose solution (ACD)(Sigma-Aldrich) and separated on a Ficoll-Histopaque density gradient (Histopaque®-1077 Hybri-Max™, Sigma-Aldrich) at 400 g for 30 min at 20 °C.
2 The mononuclear cells at the middle interface layer were harvested and washed 2–4 times with HBSS containing 0.6% ACD to remove platelet clumps.
3 The harvested cells were incubated in pre-warm red cell lysis buffer (150 mM NH_4_Cl (Sigma-Aldrich), 1 mM EDTA.2H_2_0 (BDH Laboratory Supplies) and 10 mM KHCO_3_ (BDH Laboratory Supplies), pH-adjusted to 7.5 using NaOH (Fisher Scientific)) for 12 min at room temperature.
4 After washing with HBSS containing 0.6% ACD, the cells were pooled into cold MACs buffer (0.5% Bovine serum albumin (Sigma-Aldrich), 0.6% Citrate phosphate dextrose (CPD) (Sigma-Aldrich) in 1x PBS (Sigma-Aldrich)) and spun down at 400 g for 5 min at 20 °C.
5 After centrifugation and supernatant was discarded, 500 μl MACs buffer, 100 μl MACS-Fc blocking agent and 100 μl MACS-CD34 beads for up to 10^8^ cells were added to the cell pellets. The cell suspension were then incubated at 4 °C for 30 min with constant mixing (on a roller).
6 The cells were applied to a LS column. The column was washed with cold MACS buffer and the cells were also eluted with cold MACS buffer before applying to a MS column. Finally, a total of 1.5 ml MACS buffer was passed through the MS column and the CD34+ cells were eluted in 1 ml MACS buffer.

### Erythroid differentiation of CD34+ cells

The CD34+ cells were cultured using the 3-stage erythroid culture system. During the first 8 days the cells were maintained in Basic medium which was Iscove’s medium (Biochrom) containing 3% (v/v) human AB serum (Sigma-Aldrich), 2% fetal calf serum (Hyclone, Fisher Scientiﬁc, Ltd), 10 μg/ml insulin (Sigma-Aldrich), 3 U/ml heparin (Sigma-Aldrich), 3 U/ml EPO (Roche), 200 μg/ml transferrin (R&D Systems) and 1 U/ml penicillin/streptomycin (Sigma-Aldrich) supplemented with 10 ng/ml SCF (R&D Systems) and 1 ng/ml IL-3 (R&D Systems) (primary medium). IL-3 and SCF were withdrawn from the medium on day 8 (secondary medium) and 11 (tertiary medium), respectively. In addition, extra transferrin was added to the medium to the final concentration of 500 μg/ml from day 11 onward. The cells were also cultured in the culture media without insulin and/or heparin as indicated. Cells were counted and medium was added every other day. The cultured cells were maintained at 37 °C, 5% CO_2_ throughout the culture period. At indicated time points, aliquots of cells were collected for morphological analysis using cytospin and Leishman staining. Two-sample equal variance *t*-test was carried out to determine the statistical significances of the differences in cell numbers and cell types in the original *versus* the modified culture.

## Results

### Heparin does not alter erythroblast proliferation in culture

**Fig. 1:**
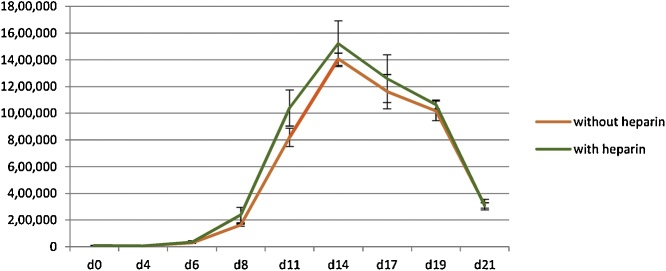
Comparison of the numbers of erythroid cells maintained in the culture media with heparin or without heparin (mean ± SD; n = 3, p > 0.05 as analyzed by Student’s *t*-test).

To study the effect of heparin on the proliferation of erythroblasts, the isolated CD34+ haematopoietic stem cells from adult peripheral blood (isolated from leukocyte-reduction system cones) were divided into 2 groups. Aliquots of approximately 10^4^ CD34+ cells were maintained in the media of a 3-stage erythroid culture system containing heparin or without heparin. The numbers of erythroid cells were evaluated every other day. There was no difference in the number of cells between the group maintained in the media without heparin and those with heparin (Fig. 1, n = 3, p > 0.05 at every time point).

### Insulin does not alter erythroblast proliferation in culture

**Fig. 2:**
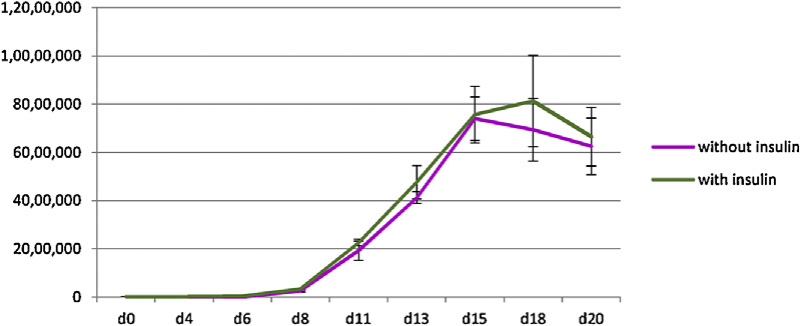
Comparison of the numbers of erythroid cells maintained in the culture media with insulin or without insulin (mean ± SD; n = 3, p > 0.05 as analyzed by Student’s *t*-test).

Next the effect of insulin on the proliferation of erythroblasts was studied. The isolated CD34+ haematopoietic stem cells from adult peripheral blood were divided into 2 groups similar to in the previous experiment. The CD34+ haematopoietic stem cells were maintained in the media with or without insulin. Similar to the previous study, there was no difference in the number of cells between the two groups at every time point (Fig. 2, n = 3, p > 0.05).

### Heparin and insulin are not required for the erythroid culture system

**Fig. 3:**
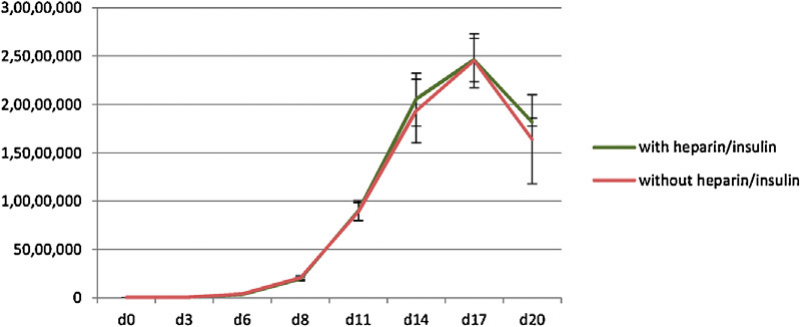
Comparison of the numbers of erythroid cells maintained in the culture media with or without heparin and insulin (mean ± SD; n = 3, p > 0.05 as analyzed by Student’s *t*-test).

Due to the observation in Fig. 1, Fig. 2, further experiment was then performed to determine whether both heparin and insulin are required for the erythroid culture system. The isolated CD34+ haematopoietic stem cells were divided into 2 groups. The first group was maintained in the media with heparin and insulin, whereas the other group was maintained in the media without both insulin and heparin. The cultures were taken through the 3-stage erythroid culture system with number of cells evaluated every other day. There was no difference in the number of cells between the two groups (Fig. 3, n = 3, p > 0.05 at every time point).

**Fig. 4:**
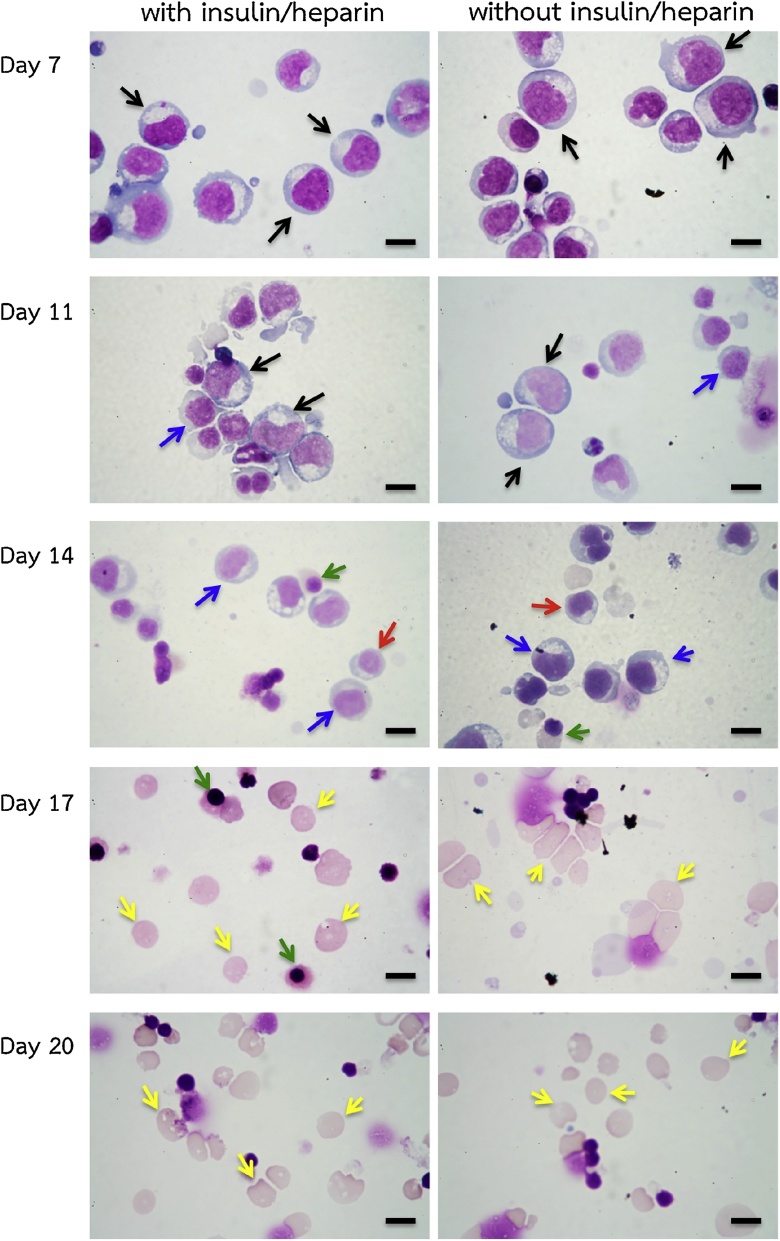
Erythroid cells maintained in the media with and without heparin and insulin stained with Leishman reagent and analyzed by light microscopy (scale bars 10 μm). Black arrows indicate proerythroblasts; blue arrows indicate basophilic erythroblasts; red arrows indicate polychromatic erythroblasts; green arrows indicate orthochromatic erythroblasts; yellow arrows indicate reticulocytes (These images are representative of three cultures).

The morphology of cultured cells was analyzed by cytospin and Leishman staining throughout the culture. There was no significant difference in morphology of cells between both groups (Fig. 4 and Table 1) with similar percentages of enucleation obtained on day 20: 90.27% ± 1.12% for the control group and 89.70% ± 1.21% for the group maintained in the media without insulin and heparin (mean ± SD, n = 3, p > 0.05 as analyzed by Student’s *t*-test).

**Table 1:** Morphological analysis of erythroid cells at different time points maintained in the media with and without heparin and insulin (mean ± SD, n = 3).

Day	Insulin/Heparin +/−	Proerythroblasts	Basophillic erythroblasts	Polychromatic erythroblasts	Orthochromatic erythroblasts	Reticulocytes
Day 8	+	68.4% ± 2.5%	27.7% ± 1.3%	3.9% ± 1.2%	0%	0%
	–	71.2% ± 2.3%	26.4% ± 1.4%	2.5% ± 0.9%	0%	0%
Day 11	+	43.7% ± 3.5%	17.4% ± 2.5%	22.1% ± 2.5%	13.5% ± 1.5%	3.3% ± 1.1%
	–	46.4% ± 2.8%	23.7% ± 2.4%	18.2% ± 2.9%	8.4% ± 1.4%	3.3% ± 0.7%
Day 14	+	4.8% ± 1.3%	20.6% ± 1.7%	30.1% ± 2.8%	28.6% ± 3.9%	15.9% ± 1.2%
	–	3.1% ± 1.1%	19.2% ± 1.6%	27.8% ± 2.1%	29.5% ± 3.8%	20.4% ± 4%
Day 17	+	0%	0%	0.7% ± 1.2%	28.8% ± 1.8%	70.5% ± 2.2%
	–	0%	0%	1% ± 1.7%	29.3% ± 2.4%	69.7% ± 1.7%
Day 20	+	0%	0%	0%	9.7% ± 1.1%	90.3% ± 1.1%
	–	0%	0%	0%	10.3% ± 1.2%	89.7% ± 1.2%

## Discussion

Several culture systems have been established for the generation of red blood cells *in vitro*. These culture systems require a number of cytokines and growth factors, which results in high cost of production. Some factors used in erythroid culture system are common among these cultures including stem cell factor, erythropoietin and transferrin [2,3,6,7], which indicates that they are necessary for erythroid differentiation. However, some factors such as insulin and heparin are used only in some culture systems [2,3,6] and these two factors are present in our culture media. As we are using this culture to study haematopoiesis due to its ability to achieve up to 95% enucleation rate, we aimed to optimize it to reduce the cost. Interestingly, we have observed in this study that heparin and insulin, which have been believed to promote erythroid proliferation and maturation [[11], [12], [13], [14], [15]], are not required for our culture system. Therefore it can be omitted if this culture system is selected for erythroid differentiation from adult haematopoietic stem cells. Since haematopoitic stem cells from different individuals have variation in their proliferation capacity, the numbers of cells (y-axis) obtained from different experiments (Fig. 1, Fig. 2, Fig. 3) in this study were slightly different. However, within the same experiment we show comparable expansion between different conditions when using haematopoitic stem cells from the same individuals. This information could then be useful for the development of *in vitro* erythroid culture system because the high cost of production is one of the limitations of this process.
